# Plasma Levels of Dimethylarginines in Preterm Very Low Birth Weight Neonates: Its Relation with Perinatal Factors and Short-Term Outcome

**DOI:** 10.3390/ijms16010019

**Published:** 2014-12-23

**Authors:** Rob M. Moonen, Maurice J. Huizing, Giacomo Cavallaro, Gema E. González-Luis, Pilar Bas-Suárez, Jaap A. Bakker, Eduardo Villamor

**Affiliations:** 1 Department of Pediatrics, Atrium Medical Center Parkstad, Heerlen 6401CX, The Netherlands; E-Mail: rmn03@atriummc.nl; 2 Department of Pediatrics, Maastricht University Medical Center (MUMC+), Cardiovascular Research Institute Maastricht (CARIM), Maastricht 6202AZ, The Netherlands; E-Mail: m.huizing@mumc.nl; 3 Neonatal Intensive Care Unit, Department of Clinical Sciences and Community Health, Fondazione IRCCS Cà Granda Ospedale Maggiore Policlinico, Università degli Studi di Milano, Milan 20122, Italy; E-Mail: giacomo.cavallaro@mangiagalli.it; 4 Department of Pediatrics, Hospital Universitario Materno-Infantil de Canarias, Las Palmas de Gran, Canaria 35016, Spain; E-Mails: gematoya@hotmail.com (G.E.G.-L.); pilybas@yahoo.es (P.B.-S.); 5 Department of Clinical Genetics, Maastricht University Medical Center (MUMC+), Maastricht 6202AZ, The Netherlands; E-Mail: j.a.bakker@lumc.nl; 6 Department of Clinical Chemistry and Laboratory Medicine, Leiden University Medical Center, Leiden 2333ZA, The Netherlands

**Keywords:** dimethylarginine, arginine, preterm infants, nitric oxide, preeclampsia, chrorioamnionitis, respiratory distress

## Abstract

Endogenously produced inhibitors of nitric oxide (NO) synthase, in particular asymmetric dimethylarginine (ADMA), are currently considered of importance in various disease states characterized by reduced NO availability. We investigated the association between plasma levels of ADMA, symmetric dimethylarginine (SDMA), l-arginine, and citrulline and perinatal factors and outcome in 130 preterm (gestational age ≤30 weeks) very low birth weight (VLBW, <1500 g) infants. Plasma samples were collected 6–12 h after birth. We did not find significant correlations between ADMA, SDMA, l-arginine, and citrulline levels and gestational age or birth weight. However, the arginine:ADMA ratio (AAR, a better indicator of NO availability than either arginine or ADMA separately) was positively correlated with gestational age. ADMA and arginine levels were not significantly different between males and females but males showed a negative correlation between ADMA levels and gestational age. Perinatal factors such as preeclampsia, chrorioamnionitis, prolonged rupture of membranes, or form of delivery did not significantly alter dimethylarginine levels or AAR. In contrast, the AAR was significantly reduced in the infants with respiratory distress, mechanical ventilation, and systemic hypotension Therefore, our data suggest that altered NO availability may play a role in the respiratory and cardiovascular adaptation in preterm VLBW infants.

## 1. Introduction

Nitric oxide (NO), a gaseous free radical, plays a central regulatory role in a variety of physiological and pathological processes, including cardiovascular and pulmonary regulation, neuronal signal transmission, inflammation, and host defense [1,2,3,4]. NO is endogenously synthesized through the action of the NO synthase (NOS) family of enzymes. Three NOS isoforms have been identified sharing a 50%–60% homology; two constitutive, the neuronal (nNOS, type I) and endothelial (eNOS, type III) enzymes, and one inducible (iNOS, type II) [4,5]. It is now appreciated that eNOS is found in other cells and tissues besides the endothelium, iNOS is found constitutively in some tissues, and there are inducible forms of both eNOS and nNOS [6]. The NO-generating reaction requires l-arginine, NADPH and oxygen as substrates, and tetrahydrobiopterin (BH_4_), thiol, flavin adenine dinucleotide, and flavin mononucleotide as cofactors. In addition to NO, the NOS-catalyzed reaction produces citrulline and NADP as co-products [6,7].

Under a number of pathological conditions, such as reduced availability of the substrate l-arginine or the cofactor BH_4_, NOS enzymatic activity becomes uncoupled, resulting in the production of superoxide (O_2_^•−^) instead of NO. Therefore, changes in NO bioavailability may be caused by: (1) alterations in the expression or activity of NOS; (2) uncoupling of NOS to produce superoxide (O_2_^•−^); or (3) degradation of NO by reacting with O_2_^•−^ from other enzymatic sources resulting in the formation of peroxynitrite (ONOO^−^) [7].

Endogenous NOS inhibitors are important modulators of the activity of the enzyme [1,2,3]. Through the action of protein arginine-*N*-methyltransferases, l-arginine can be methylated to form monomethyl-l-arginine (l-NMMA), asymmetric dimethylarginine (ADMA), and symmetric dimethylarginine (SDMA). l-NMMA and ADMA are inhibitors of all three NOS isoforms, whereas SDMA does not directly inhibit NOS but appears to interfere with the cellular transport of l-arginine, which could contribute to a reduction in intracellular substrate availability [1,2,3]. Approximately 20% of ADMA is excreted by the kidneys, whereas the other 80% is metabolized by two dimethylarginine dimethylaminohydrolases (DDAH-1 and 2) to l-citrulline and dimethylamine. Interestingly, NO overproduction itself may regulate ADMA levels through a negative feedback loop by inhibiting the activity of DDAH. SDMA is exclusively eliminated by renal excretion. [2,3,8]. Substantial experimental and clinical evidence shows that even small modifications of methylated arginine levels in particular of the l-arginine:ADMA ratio (AAR) are an important determinant of NO production and play a key role in the pathophysiology of numerous cardiovascular, pulmonary, neurological, metabolic, renal, infectious, and inflammatory conditions [1,2,3,9,10,11,12,13,14,15].

NO is also a key mediator in embryonic, fetal, and perinatal homeostasis and (patho)physiology. NO regulates embryo development, implantation, and trophoblast invasion [16]. Furthermore, vascular tone in the placenta is controlled by several vasoactive mediators, of which NO is the most important [16]. There is also growing evidence of the role of methylated arginines in normal and compromised pregnancies [2] and several perinatal conditions [17]. Very low birth weight (VLBW, <1500 g) infants are highly vulnerable in the perinatal period. Oxidative and nitrosative stress are frequent in these infants and lead to a reduction of NO bioavailability [18]. However, the information on the relationship between dimethylarginines and common perinatal diseases in VLBW infants has been scarcely investigated. The primary focus of this study was to evaluate whether ADMA and SMDA levels, at 6–12 h of life, correlate with specific perinatal/neonatal risk factors and short-term outcomes in a group of 130 preterm (≤30 weeks of gestation) VLBW infants. We also performed a systematic review of the available literature to summarize the state of knowledge about dimethylarginines and perinatal conditions.

## 2. Results and Discussion

### 2.1. Levels of Arginine, Citrulline and Dimethylarginines

In total, 130 preterm infants were enrolled in this study (32 in Maastricht, 64 in Las Palmas de Gran Canaria and 34 in Mantova). The demographic and clinical characteristics of the patients are summarized in Table 1.

No significant differences among the three centers were observed in plasma ADMA, SDMA and citrulline concentrations (Table 2). In contrast, the plasma l-arginine concentrations were significantly higher in the samples of infants from Maastricht compared to those from Las Palmas de Gran Canaria, so that the AAR was significantly higher in the patients of Maastricht than in the patients of Las Palmas de Gran Canaria (Table 2).

Gestational age and birth weight had no correlation with ADMA, SDMA, l-arginine, or citrulline concentrations (Table 3). However, the AAR showed a significant positive correlation with gestational age (Spearman’s rank correlation coefficient, ρ_s_ = 0.205; *p* = 0.020). Although ADMA, SDMA, l-arginine, and citrulline concentrations and ratios were not significantly different between males and females (Table 4), when the correlations of these values with gestational age and birth weight were separately analyzed in the two sexes, some significant results were identified. In male infants, ADMA levels and the ADMA:SDMA ratio were negatively correlated with gestational age, whereas citrulline levels were negatively correlated with birth weight (Table 3). In female infants, SDMA levels were positively correlated with gestational age, whereas ADMA, SDMA, and arginine levels were positively correlated with birth weight (Table 3).

**Table 1:** Demographic characteristics and outcomes of the included infants.

Characteristic	Mean (SD), Median [range], or *n* (%)	Number of Observations
Gestational age (weeks)	28.1 (SD 1.6)	129
Birth weight (g)	1028 (SD 244)	130
Male sex	78 (60.0)	130
Prenatal steroids	102 (80.3)	127
Preeclampsia	25 (19.2)	130
Clinical suspicion of AIS	30 (23.1)	128
Prolonged rupture of membranes	21 (16.2)	130
C-section	86 (66.2)	130
Apgar score at 1 min	6.0 [4.5–8.0]	129
Apgar score at 5 min	8.0 [7.0–9.0]	129
Respiratory distress syndrome	82 (63.1)	130
Mechanical ventilation	84 (64.6)	128
Chronic lung disease	35 (26.9)	127
Hypotension	58 (44.6)	129
Sepsis	70 (53.8)	129
NEC (Bell stage ≥2a)	6 (4.6)	130
Intracranial hemorrhage	40 (30.8)	129
Periventricular leukomalacia	10 (7.7)	129
Patent ductus arteriosus	51 (39.2)	129
Retinopathy of prematurity	15 (11.5)	125
Death before discharge	19 (14.6)	130

The number of observations represents the total number of individuals with nonmissing data for a given variable. AIS: amniotic infection syndrome; NEC: necrotizing enterocolitis.

Table 4 summarizes the effects perinatal factors on plasma ADMA, SDMA, l-arginine, and citrulline concentrations. Statistical analysis was corrected for sex, gestational age, birth weight, and center of sampling. Infants whose mother received a partial course of prenatal steroids showed a significant increase in plasma ADMA and l-arginine concentrations (Table 4). However, as these changes were in the same direction, the AAR was not significantly affected by the prenatal corticosteroids. None of the other perinatal factors analyzed—preeclampsia, clinical suspicion of amniotic infection syndrome (AIS), prolonged rupture of membranes (PROM) and vaginal delivery—significantly affected the plasma concentrations of ADMA, SDMA, l-arginine, or citrulline.

**Table 2:** Dimethylarginines, arginine, and citrulline levels and ratios in very low birth weight infants per center of sampling.

Center of Sampling	ADMA	SDMA	Arginine:ADMA	Arginine	Citrulline
Las Palmas de Gran Canaria, Spain (*n* = 64)	1.00 (0.47) ^a^	1.23 (0.51) ^a^	41.20 (25.87) ^a^	37.08 (25.17) ^a^	22.75 (8.32) ^a^
Mantova, Italy (*n* = 34)	0.92 (0.36) ^a^	1.38 (0.46) ^a^	52.18 (34.48) ^a,b^	45.24 (23.10) ^a,b^	21.79 (6.34) ^a^
Maastricht, The Netherlands (*n* = 32)	0.80 (0.32) ^a^	1.29 (0.57) ^a^	73.85 (32.02) ^b^	55.78 (26.12) ^b^	20.91 (9.16) ^a^
Total study group (*n* = 130)	0.93 (0.41)	1.29 (0.51)	52.11 (32.46)	43.82 (25.85)	22.05 (8.05)

Results of plasma concentrations (µmol/L) are expressed as mean (SD). ADMA: asymmetric dimethylarginine; SDMA: symmetric dimethylarginine. Values without a common letter (^a,b^) are significantly different (*p* < 0.05).

**Table 3:** Spearman’s Rank Order Correlation between gestational age and birth weight and dimethylarginine, arginine, citrulline levels and ratios in very low birth weight infants.

	ADMA		SDMA		ADMA:SDMA		Arginine:ADMA		Arginine		Citrulline	
	ρ_s_	*p*	ρ_s_	*p*	ρ_s_	*p*	ρ_s_	*p*	ρ_s_	*p*	ρ_s_	*p*
**Gestational age**												
Total group (*n* = 130)	−0.099	0.266	0.055	0.534	−0.172	0.051	0.205	0.020	0.155	0.080	−0.054	0.546
Males (*n* = 78)	−0.287	0.011	−0.086	0.455	−0.285	0.011	0.200	0.079	0.080	0.488	−0.099	0.388
Females (*n* = 52)	0.140	0.321	0.299	0.031	−0.053	0.710	0.133	0.347	0.203	0.150	0.020	0.886
**Birth weight**												
Total group	0.055	0.531	0.049	0.577	0.047	0.597	0.124	0.158	0.150	0.089	−0.107	0.225
Males	−0.172	0.132	−0.138	0.229	−0.012	0.917	0.097	0.396	−0.006	0.961	−0.285	0.011
Females	0.406	0.003	0.400	0.003	0.138	0.330	0.118	0.406	0.355	0.010	0.140	0.321

ADMA: asymmetric dimethylarginine; SDMA: symmetric dimethylarginine; ρ_s_ = Spearman’s rank correlation coefficient.

**Table 4:** Effects of sex and perinatal factors on dimethylarginines, arginine, citrulline levels and ratios in very low birth weight infants.

	ADMA	*p*	SDMA	*p*	ADMA:SDMA	*p*	Arginine:ADMA	*p*	Arginine	*p*	Citrulline	*p*
**Sex**												
Male (*n* = 78)	0.96 (0.43)	0.292	1.34 (0.55)	0.123	0.76 (0.29)	0.938	51.78 (34.84)	0.974	43.24 (23.40)	0.654	22.77 (8.70)	0.171
Female (*n* = 52)	0.88 (0.38)		1.20 (0.43)		0.75 (0.25)		52.61 (28.84)		44.67 (29.38)		20.96 (6.90)	
**Prenatal steroids**												
No (*n* = 26)	0.80 (0.45)	0.002 ^§^	1.23 (0.45)	0.167 ^§^	0.67 (0.25)	0.008 ^§^	55.25 (29.38)	0.138 ^§^	36.52 (17.68)	<0.001 ^§^	23.20 (6.73)	0.706 ^§^
Partial course (*n* = 17)	1.18 (0.37)		1.43 (0.45)		0.88 (0.36)		69.23 (36.06)		73.06 (28.64)		23.65 (9.97)	
Full course (*n* = 85)	0.92 (0.40)		1.29 (0.54)		0,75 (0.25)		48.54 (32.09)		40.96 (23.69)		20.94 (7.31)	
**Preeclampsia**												
No (*n* = 105)	0.95 (0.42)	0.702	1.29 (0.54)	0.911	0.77 (0.27)	0.855	49.74 (30.23)	0.859	42.91 (25.81)	0.997	22.41 (8.20)	0.338
Yes (*n* = 25)	0.86 (0.39)		1.28 (0.36)		0.68 (0.25)		62.06 (39.68)		47.60 (26.20)		20.52 (7.33)	
**AIS**												
No (*n* = 98)	0.95 (0.44)	0.119	1.31 (0.48)	0.326	0.74 (0.27)	0.901	52.76 (33.45)	0.573	45.44 (27.23)	0.483	22.69 (8.69)	0.037
Yes (*n* = 30)	0.87 (0.34)		1.23 (0.62)		0.77 (0.27)		51.46 (30.05)		39.40 (21.20)		19.80 (5.32)	
**PROM**												
No (*n* = 109)	0.93 (0.43)	0.921	1.27 (0.48)	0.939	0.75 (0.27)	0.669	51.20 (33.21)	0.907	42.66 (26.70)	0.850	22.51 (8.22)	0.156
Yes (*n* = 21)	0.94 (0.34)		1.35 (0.67)		0.75 (0.25)		56.84 (28.51)		49.81 (20.40)		19.62 (6.77)	
**Vaginal delivery**												
No (*n* = 86)	0.91 (0.41)	0.960	1.32 (0.47)	0.251	0.71 (0.26)	0.072	53.94 (35.52)	0.767	44.07 (27.98)	0.781	22.95 (8.60)	0.096
Yes (*n* = 44)	0.97 (0.43)		1.22 (0.58)		0.83 (0.26)		48.54 (25.43)		43.32 (21.38)		20.27 (6.59)	

Results of plasma concentrations (µmol/L) are expressed as mean (SD). ADMA: asymmetric dimethylarginine; SDMA: symmetric dimethylarginine; AIS: clinical suspicion of amniotic infection syndrome; PROM: prolonged rupture of membranes. Statistical analysis of sex was corrected for center of sampling, gestational age, and birth weight. All the other analyses were corrected for sex, center of sampling, gestational age, and birth weight. ^§^ Univariate analysis for linear trend.

The relationship between a number of short-term neonatal outcomes and plasma ADMA, SDMA, l-arginine, and citrulline concentrations is summarized in Table 5. No significant differences in plasma ADMA, SDMA, and citrulline concentrations were observed between the groups of infants with and without respiratory distress, mechanical ventilation, bronchopulmonary dysplasia (BPD), hypotension, sepsis, necrotizing enterocolitis (NEC), intracranial hemorrhage (ICH), periventricular leukomalacia (PVL), patent ductus arteriosus (PDA), retinopathy of prematurity (ROP), or death before discharge. Conversely, the plasma l-arginine concentration was significantly lower in the group of infants with mechanical ventilation, BPD, and hypotension (Table 5). However this reduction in l-arginine levels only affected the AAR in the infants with mechanical ventilation or hypotension but not in the infants with BPD. When the patients with hypotension were divided according to the etiology (sepsis-related or unrelated) no significant differences between the two groups were found in plasma l-arginine levels (sepsis-related hypotension: 36.00 µmol/L, SD 21.12, *n* = 41; sepsis-unrelated hypotension: 40.06 µmol/L,SD 27.49, *n* = 17) or AAR (sepsis-related hypotension: 46.18, SD 28.43; sepsis-unrelated hypotension: 46.45l, SD 27.89) Finally, the infants with respiratory distress, despite the absence of statistically significant differences in l-arginine or ADMA concentrations, showed a significant lower AAR than the infants without respiratory distress (Table 5).

### 2.2. Literature Search

The initial literature search identified 251 published studies and, ultimately, 15 were included in the systematic review. We found nine studies investigating the association between perinatal factors (fetal growth restriction, preeclampsia, cesarean section, and chorioamnionitis) and ADMA-SDMA concentrations in cord and/or neonatal blood. The main findings of these studies are summarized in Table 6. We found six studies investigating the association between neonatal factors (birth weight, prematurity, sex, mechanical ventilation, and NEC) and ADMA-SDMA concentrations in cord and/or neonatal blood. The main findings of these studies are summarized in Table 7.

### 2.3. Discussion

Prospective clinical studies have indicated a role for ADMA as a risk factor in numerous adult diseases [1,3,9,10,11,12,13,14]. Moreover, the list of clinical entities in which altered ADMA levels are found in the pediatric population continues to grow and includes conditions such as hypertension, hypercholesterolemia, chronic kidney disease or diabetes mellitus (see [17] for review). However, the information on dimethylarginines in the peri-/neonatal period is much scarce [19,20,21,22,23,24,25,26,27,28,29,30,31,32,33,34,35,36]. Herein, we analyzed the plasma concentrations of ADMA, SDMA, l-arginine, and citrulline in a cohort of 130 VLBW preterm infants. Blood samples were obtained 6–12 h after birth. We did not find any significant correlation between ADMA, SDMA, l-arginine, and citrulline levels and gestational age or birth weight. However, the AAR showed a significant positive correlation with gestational age. The AAR has been proposed to be a better indicator of NO availability than either arginine or ADMA separately because it reflects the proportion of NOS substrate and inhibitor [15]. Moreover, the AAR was significantly reduced in the infants who presented respiratory distress, received mechanical ventilation, or presented systemic hypotension. Therefore, our data suggest that altered NO availability may play a role in the respiratory and cardiovascular adaptation in preterm VLBW infants.

**Table 5:** Effects of several neonatal outcomes on dimethylarginines, arginine, citrulline levels and ratios in in very low birth weight infants.

	ADMA	*p*	SDMA	*p*	ADMA:SDMA	*p*	Arginine:ADMA	*p*	Arginine	*p*	Citrulline	*p*
**IRDS**												
No (*n* = 48)	0.86 (0.36)	0.100	1.26 (0.44)	0.624	0.70 (0.22)	0.063	59.94 (30.38)	0.018	46.44 (23.28)	0.296	21.50 (7.17)	0.535
Yes (*n* = 82)	0.97 (0.44)		1.31 (0.55)		0.78 (0.29)		47.53 (32.94)		42.28 (27.27)		22.37 (8.55)	
**BPD**												
No (*n* = 92)	0.91 (0.38)	0.981	1.28 (0.46)	0.810	0.74 (0.25)	0.805	56.47 (33.30)	0.138	47.51 (26.22)	0.030	22.09 (8.47)	0.356
Yes (*n* = 35)	0.94 (0.49)		1.33 (0.62)		0.75 (0.30)		41.58 (26.70)		33.91 (19.79)		21.80 (7.14)	
**Mechanical ventilation**												
No (*n* = 44)	0.88 (0.41)	0.569	1.24 (0.47)	0.685	0.73 (0.22)	0.865	64.11 (34.05)	0.019	51.86 (28.51)	0.039	22.52 (8.37)	0.334
Yes (*n* = 84)	0.95 (0.42)		1.30 (0.54)		0.77 (0.29)		46.05 (30.18)		39.80 (23.71)		21.93 (7.96)	
**Hypotension**												
No (*n* = 72)	0.95 (0.43)	0.452	1.26 (0.46)	0.753	0.78 (0.28)	0.130	56.83 (35.11)	0.013	49.15 (26.93)	0.001	22.29 (7.85)	0.469
Yes (*n* = 58)	0.90 (0.39)		1.33 (0.57)		0.71 (0.25)		46.26 (28.03)		37.19 (22.99)		21.74 (8.34)	
**Sepsis**												
No (*n* = 59)	0.91 (0.39)	0.855	1.25 (0.44)	0.788	0.74 (0.21)	0.710	51.85 (29.21)	0.960	43.10 (22.95)	0.975	22.15 (9.06)	0.622
Yes (*n* = 70)	0.94 (0.44)		1.32 (0.57)		0.75 (0.31)		51.69 (34.97)		43.51 (27.36)		22.01 (7.20)	
**ICH**												
No (*n* = 89)	0.91 (0.41)	0.396	1.23 (0.43)	0.101	0.76 (0.27)	0.533	53.60 (31.01)	0.625	44.47 (25.86)	0.831	22.64 (8.80)	0.114
Yes (*n* = 40)	0.98 (0.42)		1.41 (0.66)		0.74 (0.26)		48.30 (35.85)		42.15 (26.39)		20.88 (6.03)	
**PVL**												
No (*n* = 119)	0.91 (0.42)	0.683	1.29 (0.52)	0.376	0.74 (0.27)	0.099	53.98 (32.78)	0.148	45.19 (26.32)	0.116	22.04 (8.36)	0.578
Yes (*n* = 10)	1.02 (0.34)		1.16 (0.42)		0.91 (0.24)		33.34 (21.06)		28.90 (14.07)		21.30 (2.36)	
**PDA**												
No (*n* = 78)	0.89 (0.34)	0.792	1.29 (0.49)	0.825	0.73 (0.28)	0.707	56.26 (30.02)	0.931	46.59 (23.95)	0.561	22.08 (9.39)	
Yes (*n* = 51)	0.97 (0.49)		1.27 (0.54)		0.77 (0.25)		46.69 (35.05)		40.25 (28.14)		21.82 (5.44)	
**ROP**												0.526
No (*n* = 110)	0.90 (0.38)	0.752	1.26 (0.45)	0.262	0.75 (0.26)	0.205	53.62 (32.14)	0.967	45.21 (26.48)	0.225	22.22 (8.50)	0.160
Yes (*n* = 15)	0.88 (0.36)		1.40 (0.75)		0.68 (0.26)		47.70 (33.10)		33.47 (12.08)		19.67 (3.90)	
**Death before discharge**												
No (*n* = 111)	0.90 (0.40)	0.108	1.27 (0.49)	0.359	0.74 (0.27)	0.381	52.26 (31.93)	0.558	43.03 (24.65)	0.169	21.68 (8.29)	0.376
Yes (*n* = 19)	1.08 (0.46)		1.40 (0.64)		0.81 (0.25)		51.24 (36.30)		48.42 (32.44)		24.16 (6.22)	

Results of plasma concentrations (µmol/L) are expressed as mean (SD). ADMA: asymmetric dimethylarginine; SDMA: symmetric dimethylarginine; IRDS: infant respiratory distress syndrome; BPD: bronchopulmonary dysplasia; ICH: intracranial hemorrhage; PVL: periventricular leukomalacia; PDA: patent ductus arteriosus; ROP: retinopathy of prematurity. Statistical analysis was corrected for sex, center of sampling, gestational age, and birth weight.

**Table 6:** Summary of studies describing the association between perinatal factors and dimethylarginines in newborn infants.

Study	Perinatal Factor	Target Group (Number)	Blood Sample	Findings
Tsukahara *et al*. (2008) [37]	Preeclampsia, chorioamnionitis, abruptio placentae, delivery mode	Preeclampsia (*n* = 11), chorioamnionitis (*n* = 2), abruptio placentae (*n* = 2), control group (*n* = 33, 8 born vaginally and 25 by cesarean)	Umbilical cord	ADMA levels were independent of the delivery mode and maternal preeclampsia.
Braekke *et al*. (2009) [21]	Preeclampsia	Preeclampsia (*n* = 47), control (*n* = 51), preterm and term infants	Maternal, umbilical cord	Elevated ADMA, SDMA and l-arginine levels in women with preeclampsia. Elevated SDMA levels in newborns from preeclamptic pregnancies. Maternal ADMA < neonatal ADMA.
Alacam *et al*. (2011) [19]	Preeclampsia	Preterm newborns from preeclamptic pregnancies (*n* = 21), term newborn from normal pregnancies (*n* = 28)	Umbilical cord	Elevated ADMA levels and decreased. AAR in newborns from preeclamptic pregnancies.
Tamás *et al*. (2013) [25]	Preeclampsia	Preterm newborns with maternal preeclampsia (*n* = 16), term newborns with (*n* = 11) or without (*n* = 14) maternal preeclampsia	Maternal, umbilical cord
Chedraui *et al*. (2013) [22]	Preeclampsia	GA >36 weeks, preeclampsia (*n* = 31), control (*n* = 31)	Umbilical cord	Preeclampsia did not affect neonatal ADMA levels but increased NO plasma levels (determined by nitrite assay).
Kul *et al*. (2009) [23]	Maternal hypertension, gestational diabetes, smoking during pregnancy, meconium staining	Maternal hypertension (*n* = 14), gestational diabetes (*n* = 11), smoking during pregnancy (*n* = 13), meconium staining (*n* = 13), control group (*n* = 51)	Maternal, umbilical cord	Maternal ADMA < neonatal ADMA in all groups. Newborns with meconium staining showed higher ADMA levels than the other groups.
Pisaneschi *et al*. (2012) [24]	Fetal growth restriction	Term newborns, AGA (*n* = 40), term newborns, SGA (*n* = 20), preterm newborns, SGA (*n* = 15), DCDA twins with discordant fetal growth (*n* = 12)	Umbilical cord, heel sampling, at birth, 24 and 72 h	Decreased ADMA levels and increased NO production and in infants with fetal growth restriction.
Vida *et al*. (2012) [26]	Delivery mode	Full term, vaginal delivery (*n* = 10), cesarean section (*n* = 20)	Umbilical cord, venous (day 2)	After cesarean section, ADMA levels in umbilical artery remained elevated and fell postnatally at a slower rate than after vaginal birth.
Alfiero Bordigato *et al*. (2011) [20]	Chorioamnionitis	ELBW infants, chorioamnionitis (*n* = 14), no chorioamnionitis (*n* = 15)	Arterial (birth, day 3 and day 5–7)	Elevated ADMA levels (at birth and through the first week of life) in infants born to mothers with histologic chorioamnionitis.

AGA: appropriate for gestational age; SGA: small for gestational age; LGA: large for gestational age; DCDA: dichorionic diamniotic; ELBW: extremely low birth weight (<1000 g); ADMA: asymmetric dimethylarginine; SDMA: symmetric dimethylarginine; AAR: Arginine:ADMA ratio; NO: nitric oxide.

**Table 7:** Summary of studies describing the association between neonatal outcome and dimethylarginines in cord blood or plasma of infants.

Study	Neonatal Characteristic/Outcome	Target Group (Number)	Blood Sample	Findings
Mittermayer *et al.* (2006) [31]	Gestational age, birth weight, sex	Preterm male newborns (*n* = 11), preterm female newborns (*n* = 8), term born males (*n* = 10), term born females (*n* = 11)	Umbilical cord	ADMA levels preterm > term. ADMA levels preterm male > preterm female. Birth weight correlated negatively with ADMA in preterm infants and positively in term infants.
Tsukahara *et al*. (2008) [37]	Gestational age, birth weight, sex	Preterm infants (*n* = 11), term infants (*n* = 22)	Umbilical cord	ADMA levels inversely correlated with gestational age and birth weight. No male/female differences.
Vida *et al.* (2009) [35]	Gestational age, birth weight, sex, several neonatal conditions (infection, respiratory and cardiovascular morbidity, asphyxia)	ELBW infants (*n* = 20)	Venous (on day 3, 7, 14, 21 and 28)	ADMA levels and AAR increased with postnatal age. Negative correlation between ADMA levels and gestational age, dopamine-need on the 3rd day of life and late infection. Positive correlation between ADMA levels and birth weight.
Richir *et al.* (2007) [33]	Necrotizing enterocolitis	VLBW infants with NEC (*n* = 10), VLBW infants without NEC (*n* = 10)	Venous (at the moment of diagnosis)	ADMA and AAR were decreased in infants with NEC.
Moonen *et al.* (2010) [32]	Necrotizing enterocolitis	VLBW infants with NEC (*n* = 5), VLBW infants without NEC (*n* = 123)	Venous (6–12 h after birth)	ADMA and AAR were similar in infants with or without NEC.
Richir *et al.* (2008) [34]	Mechanical ventilation	VLBW infants with mechanical ventilation (*n* = 15), VLBW infants without mechanical ventilation (*n* = 15)	Umbilical cord	ADMA levels elevated in infants who required mechanical ventilation. ADMA concentration was significantly related to length of mechanical ventilation.

ELBW: extremely low birth weight (<1000 g); VLBW: very low birth weight (<1500 g); ADMA: asymmetric dimethylarginine; SDMA: symmetric dimethylarginine; AAR: Arginine:ADMA ratio. NEC: necrotizing enterocolitis.

Newborn infants have substantially elevated concentrations of ADMA when compared with children and adults [38]. Moreover, ADMA levels increase during the first weeks of life [35]. In addition, several studies reported that umbilical plasma levels of ADMA were elevated in preterm infants when compared with term infants [31,37,39]. It should be noted that the sample size in the above studies was very small (see Table 7). Herein, we report the largest cohort of preterm infants in which dimethylarginine levels have been assessed. As mentioned above, we found that gestational age and birth weight did not correlate with arginine or dimethylarginine levels. However, the AAR was positively correlated with gestational age. Therefore, the more preterm infants tended to have lower arginine levels and higher ADMA levels. Unfortunately, with our data we cannot answer the question whether dimethylarginine concentrations are higher in preterm than in term infants. The absence of a group of term infants is a limitation of our study. Nevertheless, the fetal−neonatal period appears to be the moment of life with the highest concentrations of ADMA. Paradoxically, NO production is elevated in the perinatal period, particularly in preterm infants [39,40]. The possible biological significance of this fact remains unknown.

When compared with females, males are at higher risk of prematurity as well as pulmonary, neurological, gastrointestinal, and cardiovascular prematurity-related conditions. This “male disadvantage” with respect to neonatal morbidity and mortality has been recognized for more than three decades but the contributing biological mechanisms are poorly understood and likely to be multifactorial [41]. Interestingly, Mittermayer *et al*. observed that ADMA levels were higher in male than in female preterm infants [31]. In contrast, Richir *et al*. [33] and Tsukahara *et al*. [37] did not find sex-related differences in ADMA levels in preterm infants. Accordingly, we found that ADMA, SDMA, l-arginine, and citrulline concentrations, as well as AAR and ADMA:SMDA ratio, were not significantly different between males and females. Nevertheless, we found some sex-dependent correlations between arginine/dymethylarginine levels and gestational age and birth weight (Table 3). The most relevant was the negative correlation between ADMA levels and gestational age, which was observed in male infants. The reason for this correlation remains to be clarified, but might be a consequence of increased ADMA synthesis, decreased metabolism by DDAH, decreased clearance by immature kidneys, or some combination of those factors [37]. Interestingly, in the male infants, the ADMA:SDMA ratio was negatively correlated with gestational age. The ADMA:SDMA ratio is accepted as a rough estimate of the DDAH activity, a high ratio being suggestive of low activity [42]. Therefore, our data suggest a different pattern of maturation of DDAH activity between male and female preterm infants. It is becoming increasingly apparent that DDAH activity is regulated by NO through *S*-nitrosylation and that the activity of NOS is controlled by DDAH through metabolization of ADMA and the consequent modulation of its levels [8,37]. Remarkably, we observed that females had a significant positive correlation between birth weight and the levels of ADMA, SDMA and arginine. Therefore, our data suggest the presence of sex-related differences in ADMA metabolism and/or excretion in VLBW infants and it could be speculated a role for elevated ADMA levels in the male disadvantage of these neonates.

Preeclampsia is the pregnancy condition in which NO homeostasis has been more intensively studied. Although preeclampsia is characterized by maternal and feto-placental endothelial dysfunction, data on circulating NO in this condition are inconsistent [43]. The severity of the disease or gestational age at sampling might explain these variations. Nevertheless, concentrations of cyclic GMP are consistently lower in preeclampsia indicating decreased NO bioactivity [43]. This is in line with increasing maternal plasma ADMA during and before the onset of preeclampsia as compared with healthy pregnancy [2,43,44]. On the other hand, preeclampsia did not appear to produce similar effects in the newborn. Thus, in our cohort, and in other studies [21,22,25], there were no significant alterations in ADMA concentration at birth in the infants of preeclamptic mothers. In contrast, Alacam *et al*. reported elevated ADMA levels in preterm newborns from preeclamptic pregnancies [19]. However, these infants were compared with a group of infants born at term. Therefore, the elevated ADMA levels could have been related to the prematurity and not to the preeclampsia.

Changes in neonatal ADMA levels have been correlated with other perinatal conditions such as fetal growth restriction [24], chrorioamnionitis [19] and delivery mode. Our results do not confirm those findings. As mentioned above, the only perinatal factor that altered ADMA levels in our cohort was the exposure to a partial course of antenatal steroids. Interestingly, Tain *et al*. observed that administration of dexamethasone to pregnant rats increased offspring ADMA levels [45]. In contrast, and in agreement with our observations, AAR remained unchanged because prenatal corticosteroids also augmented l-arginine levels [45]. Although antenatal corticosteroids have become standard therapy for women in whom preterm delivery is threatened, debate remains on its long-term effects into adulthood. In experimental models, corticosteroid-exposed offspring are characterized by hypertension, and reduced NO bioavailability has been proposed as a major component of this phenomenon [45,46]. Nevertheless, our observation on the effects of antenatal corticosteroids on ADMA levels was limited to the infants exposed to a partial course. It should be noted these infants may present a different clinical profile than infants who received a complete course. For example, the presence of an extremely imminent preterm delivery may have precluded the completion of the antenatal corticosteroid course and also have influenced the ADMA levels.

NO is an important mediator of normal lung development, pulmonary vascular resistance and ventilation perfusion matching [34,47]. Dysregulation of the arginine-ADMA-NO pathway plays a crucial role in the development and/or progression of chronic respiratory diseases [47]. Herein, we found that the AAR was significantly reduced in the infants who presented respiratory distress and received mechanical ventilation. The reduction in AAR was the consequence of lower l-arginine levels since ADMA levels were not significantly affected. In contrast to our results, Richir *et al*. observed that preterm infants who required mechanical ventilation showed elevated ADMA levels compared to non-ventilated preterm infants [34]. However, the increase in ADMA levels did not significantly alter the AAR. Nevertheless, altogether these results suggest that reduced NO bioavailability may contribute to the pathogenesis of respiratory distress in preterm infants. Inhaled NO therapy might be a way to counteract the reduced availability of NO in airways and pulmonary vasculature. However, the present available evidence does not support the use of inhaled NO in preterm infants with respiratory distress and inhaled NO therapy cannot be recommended for the routine treatment of respiratory failure in these infants [48].

Hemodynamic problems are common in very preterm infants. They occur in the context of incomplete myocardial and vascular development and in cardiovascular responses to interventions which are, as a result, limited and often uncertain and unpredictable [49]. We observed that systemic hypotension was accompanied by a significant decrease in AAR. Again, this alteration in the ratio was due to diminished levels of l-arginine and not to increased levels of ADMA. Since NO is a vasodilator, it is surprising that a possible reduction in its bioavailability is accompanied by systemic hypotension. The underlying mechanisms of this paradoxical reaction are not apparent. Zoccali *et al*. reported a correlation between ADMA suppression and hypotension in adult patients with acute infections [50]. They speculate that during infectious processes plasma ADMA suppression may serve to stimulate NO synthesis [50]. When we separately analyzed the infants with suspected sepsis-related hypotension and the infants with hypotension related to other etiologies, we found similar AARs in both groups. Therefore, our data do not suggest that the altered AAR in hypotensive infants is related to infection. Bergamini *et al*. [51] and Csiky *et al*. [52] described an association between elevated ADMA levels and hypotension in adult patients subjected to hemodialysis. They speculate that excessive NO generation may induce the increase in plasma ADMA levels in order to prevent further fall in blood pressure [51,52]. We did not measure NO plasma levels or NO metabolites in the urine of our patients and, therefore, we cannot establish a correlation between hypotension and elevated NO production. Nevertheless, low numerical blood pressure is considered an unreliable marker of low systemic blood flow and circulatory compromise in very preterm infants [49]. Further studies will be needed to assess the possible role of ADMA in the circulatory adaptation and hemodynamic homeostasis of preterm infants.

Our study has several limitations that need to be addressed. First, although it is the largest study of its type, the sample size is still small. The power limitations and type II error should therefore be taken into account when interpreting the results. This is particularly valid when examining outcomes with low incidence rates. Second, we did not include a control group of healthy term infants. Third, neither maternal dimethylarginine levels nor neonatal NO or its oxidation products nitrite and nitrate were determined. Although some of these measurements are technically complex and relatively unreliable in preterm infants [34], its correlation with ADMA levels and AAR would have contributed to clarify the picture of NO bioavailability in our cohort. Finally, we performed a cross-sectional study with analysis of dimethlylarginine values at one point and this is not as valuable as a longitudinal study with several values. It should be taken into account that the alterations in ADMA levels or AAR may be only present later in life, under the disease stress conditions, when the demand for NO is potentially increased. Accordingly, Bassareo *et al*. reported that blood ADMA levels were higher in a group of former preterm newborns (birth weight <1000 g, aged 17–29 years) than in healthy controls born at term [53]. Moreover, in the same population of ex-preterm infants, a higher ADMA:SDMA ratio than in the control group was observed [54]. These data indicate that alterations in the arginine−ADMA−NO pathway persist in adulthood and suggest that extreme prematurity at birth may underlie the development of a circulatory dysfunction, predictive of potential future adverse cardiovascular events [53,54].

## 3. Experimental Section

### 3.1. Patients and Study Design

This study was performed in a cohort of 130 preterm infants admitted to the level III neonatal intensive care unit of the Maastricht University Medical Center (Maastricht, The Netherlands), Hospital Universitario Materno-Infantil de Canarias (Las Palmas de Gran Canaria, Spain), and Carlo Poma Hospital (Mantova, Italy) between July 2007 and October 2008. All infants had been enrolled in a study on Carbamoyl Phosphate Synthetase (CPS) Polymorphisms as Risk Factor for NEC (ClinicalTrials.gov Protocol Registration System, NCT00554866) for which the patient inclusion criteria were gestational age ≤30 weeks and birth weight ≤1500 g. Written informed consent was obtained from the parents for inclusion in the above study. The use of the clinical data of the patients for the present study was approved by the Local Research Ethics Committees of the participating centers. Exclusion criteria were the following: blood transfusion, enteral or parenteral protein intake, or inhaled NO administration before blood sampling. The correlation between arginine, citrulline and dimethylarginine levels and CPS polymorphisms and NEC in the same group of patients has been the subject of another publication of our group [32]. One blood sample (500 µL) was obtained between 6 and 12 h after birth from an umbilical artery or peripheral artery catheter. When not available, the blood sample was obtained from venous puncture. Immediately after collection, heparinized blood samples were put on ice and centrifuged within 10 min (4000 rpm, 10 min, 4 °C) to obtain plasma. The plasma was deproteinized with 6 mg of solid 5-sulfosalicylic acid (SSA; Sigma, St. Louis, MO, USA) per 100 µL plasma, and stored at −80 °C until further analysis. The samples obtained in Las Palmas and Mantova were transported on dry ice to Maastricht where all the analyses were performed.

The perinatal and neonatal data of enrolled infants (Table 1) were extracted from the database of the NCT00554866 trial. Gestational age was determined by the last menstrual period and early ultrasounds (before 20 weeks of gestation). Small for gestational age was defined as birth weight for gestational age below the sex-specific tenth percentile. Clinical suspicion of AIS defined as every clinical suspicion of infection of the chorion, amnion, amniotic fluid, placenta, or a combination as judged by the obstetrician. PROM was defined as rupture of membranes >24 h before delivery. Prenatal exposure to a single course of antenatal steroids was defined as two doses of betamethasone administered 24 h apart, and exposure to a partial course of antenatal steroids was defined as administration of a single dose of betamethasone <24 h prior to delivery. Respiratory distress was defined as requirement for oxygen supplementation or respiratory support due to tachypnea, grunting, nasal flaring, retractions, or cyanosis. BPD was defined as a supplemental oxygen requirement at 36 weeks of corrected gestational age to maintain oxygen saturation >90% [55]. Arterial hypotension was defined as the need for volume expansion or inotropic support. PDA was defined as a requirement for indomethacin or ibuprofen and/or surgical ligation. A diagnosis of sepsis required signs of generalized infection, a positive blood culture and antibiotic therapy. ICH was classified by using the four-level grading system [56]. Grade <2 ICHs were not included in the analysis.

### 3.2. Plasma Amino Acid and Dimethylarginine Analysis

Concentrations of arginine, citrulline, ADMA and SDMA were determined in plasma using an ultra-performance liquid chromatography (UPLC) separation module coupled to an electrospray ionization tandem mass spectrometry (ESI-MS/MS, Quattro Premier, Waters, Etten-Leur, The Netherlands). Separation of the components of interest was adapted from the described method for the determination of amino acids [57]. Briefly, plasma samples were mixed with stable isotope labelled ADMA and deproteinized with SSA and diluted. Amino acids, ADMA and SDMA were detected in the multiple reaction mode (MRM) in ESI positive mode [58]. All analyses were performed in one laboratory (Department of Clinical Genetics, Maastricht University Medical Center, Maastricht, The Netherlands).

### 3.3. Literature Search

We searched the electronic database of Pubmed/Medline (http://www.ncbi.nlm.nih.gov/pubmed, last accessed on 12 July 2014) in order to identify all the studies that evaluated the relation of dimethylarginines, arginine and citrulline with perinatal factors and neonatal outcomes (as listed in Table 1). The search strategy was “dimethylarginine” (All Fields) and “(perinatal factor or neonatal outcome as described in Table 1)” (All Fields) and “infant” (All Fields). A second search strategy was (“arginine” (All Fields) or “citrulline” (All Fields)) AND “(neonatal outcome as described in Table 1)” (All Fields) and “infant” (All Fields). To find additional data, titles from the recovered articles were entered in the “Times Cited” function in ISI Web of Knowledge and the “Cited by” function in Google Scholar. Finally, the references of all relevant articles were examined to identify publications not retrieved by electronic search. No language restrictions were applied but all papers included in the final selection were in English.

### 3.4. Statistics

Results for continuous variables are expressed as mean (SD) or, if variables were not normally distributed, as median (interquartile range). Differences between mean values in the three centers were assessed by one-way ANOVA followed by Bonferroni’s *post hoc* *t*-test. Correlations of gestational age and birth weight with ADMA, SDMA, l-arginine, and citrulline concentrations were evaluated using Spearman’s correlation coefficient (ρ_s_). The possible relationship between ADMA, SDMA, l-arginine, and citrulline concentrations and perinatal factors and neonatal outcome was evaluated by univariate analysis of variance with adjustment for sex, gestational age, birth weight and center of sampling. Differences were considered significant at a *p* < 0.05. All analyses were performed using IBM SPSS Statistics for Windows (Version 22.0; Armonk, NY, USA).

## 4. Conclusions

In conclusion, our study does not confirm the previously reported associations between perinatal factors such as preeclampsia, chorioamnionitis, or mode of delivery and dimethylarginine levels in preterm VLBW infants. In contrast, we found a positive correlation between AAR and gestational age and an association between low AAR and the presence of systemic hypotension, respiratory distress, and mechanical ventilation requirement. Therefore, our data suggest that altered NO availability may play a role in the respiratory and cardiovascular adaptation in preterm VLBW infants. Future prospective and longitudinal studies are needed to confirm these findings and to clarify the role of NO bioavailability in peri- and neonatal (patho)physiology.
